# Revascularização Completa *Versus* Tratamento da Artéria Culpada no Infarto com Supradesnivelamento do Segmento ST: Registro Multicêntrico

**DOI:** 10.36660/abc.20180346

**Published:** 2020-08-19

**Authors:** Julia Cremona Cadore, Mariana Vargas Furtado, Rogério Tumelero, Alexandre Tognon, Ana Maria Krepsky, Denis Cadore, Karen Brasil Ruschel, Julia Caldas Bedin, Thais Conte, Carisi Anne Polanczyk

**Affiliations:** 1 Universidade Federal do Rio Grande do Sul PPG Ciências Cardiovasculares: Cardiologia Porto Alegre RS Brasil Universidade Federal do Rio Grande do Sul - PPG Ciências Cardiovasculares: Cardiologia, Porto Alegre, RS - Brasil; 2 Hospital de Clínicas de Porto Alegre Instituto de Avaliação de Tecnologia em Saúde Porto Alegre RS Brasil Hospital de Clínicas de Porto Alegre - Instituto de Avaliação de Tecnologia em Saúde, Porto Alegre, RS - Brasil; 3 Associação Hospitalar Beneficente São Vicente de Paulo Passo Fundo RS Brasil Associação Hospitalar Beneficente São Vicente de Paulo, Passo Fundo, RS - Brasil; 4 Universidade Federal do Rio Grande do Sul Faculdade de Medicina Porto Alegre RS Brasil Universidade Federal do Rio Grande do Sul - Faculdade de Medicina, Porto Alegre, RS - Brasil; 5 Hospital de Clínicas de Porto Alegre Porto Alegre RS Brasil Hospital de Clínicas de Porto Alegre, Porto Alegre, RS – Brasil

**Keywords:** Infarto do Miocárdio com Supradesnível do Segmento ST/mortalidade, Estudos de Coortes, Hemodinâmica, Registros de Óbitos, Angina Pectoris, Acidente Vascular Cerebral, Parada Cardíaca, Intervenção Coronária Percutânea

## Abstract

**Fundamento:**

São restritos os dados sobre o manejo e o prognóstico dos pacientes com infarto agudo do miocárdio com supradesnivelamento do segmento ST (IAMCSST) com acometimento multiarterial no Brasil, o que mostra a necessidade de investigar as estratégias de revascularização disponíveis.

**Objetivo:**

Avaliar os desfechos relacionados à revascularização completa em comparação com o tratamento da artéria culpada em pacientes multiarteriais com IAMCSST.

**Métodos:**

Foi realizada um estudo de coorte prospectiva em dois centros de hemodinâmica do Sul do Brasil, com seguimento de 1 ano após a intervenção índice. O desfecho primário foi composto de óbito cardiovascular, reinfarto ou angina recorrente e secundários acidente vascular encefálico, parada cardiorrespiratória não fatal, sangramento maior ou necessidade de reintervenção. A probabilidade de ocorrência de desfechos foi comparada entre os grupos através de regressão logística binária. Considerou-se como estatisticamente significativo o valor de probabilidade < 0,05.

**Resultados:**

Participaram 85 pacientes, com média de idade de 62±12 anos, sendo 61 (71,8%) do sexo masculino. Cinquenta e oito (68,2%) pacientes receberam a estratégia de revascularização completa e 27 (31,8%), a de revascularização incompleta. A chance de ocorrência tanto do desfecho primário quanto do secundário foi significativamente maior entre os indivíduos tratados com revascularização incompleta quando comparados com os tratados com estratégia completa [razão de chances (OR) 5,1, intervalo de confiança de 95% (IC95%) 1,6-16,1 vs. OR 5,2, IC95% 1,2-22,9, respectivamente], assim como os óbitos cardiovasculares (OR 6,4, IC95% 1,2-35,3).

**Conclusão:**

Dados deste registro regional, de dois centros do Sul do Brasil, demonstram que a estratégia de revascularização completa esteve associada à redução significativa dos desfechos primário e secundário no seguimento de 1 ano quando comparada à estratégia de revascularização incompleta. (Arq Bras Cardiol. 2020; 115(2):229-237)

## Introdução

O infarto agudo do miocárdio com supradesnivelamento do segmento ST (IAMCSST) constitui um problema de extrema relevância em saúde pública,^1^ apresentando alta taxa de mortalidade caso não seja tratado adequadamente.^2^ Aproximadamente 50% dos pacientes apresentam doença arterial coronariana (DAC) multiarterial,^3 - 4^ sendo o prognóstico ainda mais desfavorável.^5^

As opções terapêuticas para esse grupo mais complexo incluem intervenção coronariana percutânea (ICP) primária na artéria culpada pelo infarto (ACI) e ICP nas demais estenoses somente na presença de isquemia espontânea ou achados de risco em testes não invasivos (revascularização incompleta – RI); ICP multiarterial no momento da ICP primária (revascularização completa – RC); ICP primária na ACI e tratamento estadiado das demais estenoses (RC estadiada). Estudos iniciais demonstraram resultados conflitantes.^6^ O estudo PRAMI ( *Preventive Angioplasty in Acute Myocardial Infarction* ), no entanto, trouxe uma mudança nesse paradigma, na medida em que demonstrou o benefício da ICP multiarterial comparada com a ICP primária apenas da ACI.^7^ Outros ensaios clínicos reforçaram a hipótese de que uma estratégia de RC poderia ser benéfica e segura em pacientes selecionados com IAMCSST.^8 - 10^

Com base nesses achados, o *American College of Cardiology* (ACC) e a *American Heart Association* (AHA), em 2015, atualizaram a recomendação anterior, sendo, então, factível tanto a RC quanto a abordagem estadiada na ocasião da ICP primária em pacientes hemodinamicamente estáveis.^4^ A diretriz da Sociedade Europeia de Cardiologia (ESC), de 2017, segue a mesma orientação.^11^ Já a diretriz da Sociedade Brasileira de Cardiologia (SBC), de 2015, considera razoável o tratamento de estenose grave de menor complexidade localizada no sistema coronário relacionado ao vaso infartado.^12^ Por outro lado, salienta que estes pacientes apresentam maior propensão à ocorrência de novos eventos coronários no período de 1 ano, sugerindo, então, que as estenoses coronárias graves não relacionadas diretamente ao procedimento índice devam ser abordadas em um segundo tempo, de forma estadiada.^12^

Neste estudo, objetivamos avaliar os desfechos de vida real relacionados a RC *versus* tratamento da artéria culpada em pacientes multiarteriais com IAMCSST em dois hospitais da Região Sul do Brasil.

## Método

### Delineamento da Pesquisa

Estudo de registro, com pacientes hospitalizados por IAMCSST e DAC multiarterial em dois centros no Sul do Brasil. Foram coletados dados prospectivos no período de outubro de 2015 a março de 2016, com informações da internação hospitalar. Foram ainda coletados dados retrospectivos no período de janeiro a setembro de 2015, por revisão de prontuário médico. Os desfechos primário e secundário foram avaliados prospectivamente por contato telefônico mensal, durante 12 meses após alta hospitalar após evento índice.

### Seleção de Pacientes

Foram incluídos pacientes com idade ≥ 18 anos e de ambos os sexos, admitidos nos hospitais supracitados no período de 6 meses, com o diagnóstico de IAMCSST, tratados com ICP primária e DAC multiarterial à cineangiocoronariografia – definida como presença de lesão igual ou superior a 70% pela análise visual da angiografia em duas ou mais projeções, em mais de uma artéria coronária. Pacientes encaminhados a esses hospitais para angioplastia de resgate após o uso de trombolíticos e que apresentavam DAC multiarterial também foram elegíveis.

Foram excluídos pacientes com cirurgia de revascularização do miocárdio (CRM) prévia, choque cardiogênico na admissão, indicação de CRM após angioplastia primária, lesão no tronco da coronária esquerda, lesão na artéria descendente anterior (ADA) proximal ou na artéria circunflexa proximal ou oclusão crônica em artéria não culpada pelo infarto (ANCI), os quais, pela avaliação da equipe assistente, se beneficiariam de cirurgia de revascularização.

### Coleta de Dados

A coleta das informações pertinentes ao estudo ocorreu em formulário padronizado durante o período de hospitalização para o tratamento do evento agudo, incluindo características demográficas, exames realizados na emergência do hospital, resultado da cinecoronariografia, tratamento instituído, além dos dados de seguimento pelo período de 1 ano. As condutas adotadas durante o atendimento do paciente foram de responsabilidade da equipe assistencial, sem influência dos pesquisadores. A pesquisa se deu em conformidade com a resolução nº 466/2012 e foi aprovada pelos Comitês de Ética e Pesquisa de ambas instituições. Os pacientes incluídos prospectivamente assinaram o termo de consentimento livre e esclarecido (TCLE) após o término do exame inicial (cinecoronariografia); para a coleta de dados retrospectivos, os pesquisadores assinaram o termo de sigilo de dados.

### Seguimento e Desfechos de Interesse

A evolução e ocorrência dos desfechos intra-hospitalares foram avaliados durante a hospitalização e, posteriormente, através de busca ativa via contato telefônico e revisão de prontuário médico. O desfecho primário foi definido como a ocorrência de: (1) óbito por causa cardiovascular; (2) reinfarto – definido como recorrência da dor isquêmica (embora não seja imprescindível), nova elevação de segmento ST ≥ 0,1 mV ou nova onda Q, no mínimo em duas derivações contíguas ou valor de marcadores séricos (troponina ou CK-MB) alterados (acima do limite superior do normal conforme valor de referência do laboratório local ou pelo menos 50% acima do valor do exame anterior); ou (3) angina recorrente – definida como retorno da dor, necessidade de uso de nitrato sublingual ou reinternação por angina recorrente.

O desfecho secundário foi composto de: (1) acidente vascular encefálico (AVE); (2) parada cardiorrespiratória (PCR) não fatal; (3) sangramento maior (definido como necessidade de transfusão sanguínea por queda de mais de 3 g/dL no exame de hemoglobina, e/ou hemoglobina abaixo de 10 g/dL, e/ou instabilidade hemodinâmica, e/ou prolongamento na internação por sangramento maior, e/ou AVE hemorrágico, ou (4) necessidade de reintervenção percutânea ou cirúrgica não programada.

### Análise Estatística

A análise estatística foi realizada através do IBM SPSS Statistics, versão 22.0 para Windows. A idade e o tempo entre a ICP primária e o novo procedimento foram descritas como média ± desvio padrão. As variáveis categóricas foram descritas como frequência absoluta e relativa. As distribuições das variáveis numéricas foram comparadas entre os grupos tratados com RC ou apenas da lesão culpada pelo infarto utilizando-se análise de variância com um critério de classificação e, das variáveis categóricas, utilizando-se o qui-quadrado de Pearson com correção de continuidade ou teste exato de Fischer quando adequado. A chance de ocorrência de desfecho primário, secundário e óbito por qualquer causa foi comparada entre os grupos acima descritos utilizando-se regressão logística binária. Na análise multivariável, os modelos foram comparados entre si utilizando-se o teste da razão de verossimilhança. As curvas de Kaplan-Meier para IAMCSST foram calculadas para RC e RI. As diferenças na taxa de sobrevida foram avaliadas pelo teste log-rank. As razões de chance foram descritas com os respectivos intervalos de confiança de 95%. Considerou-se como estatisticamente significativos testes com valor de probabilidade < 0,05.

## Resultados

No período de janeiro de 2015 a março de 2016, foram admitidos nas emergências dos dois centros 248 pacientes com diagnóstico de IAMCSST; destes, 85 (34,3%) pacientes apresentavam DAC multiarterial.

Do total de 85 pacientes, 58 (68,2%) receberam estratégia de RC e 27 (31,8%) receberam estratégia de revascularização apenas da ACI. A idade média foi 62±12 anos, e 61 (71,8%) participantes eram do sexo masculino. O infarto de parede inferior ocorreu em 42 (49,4%) pacientes, seguido pelo infarto de parede anterior em 37 (43,5%). Os pacientes classificados como Killip I na admissão foram 71 (83,5%) e 67 (78,8%) apresentavam doença coronária biarterial. A ADA foi a responsável pelo infarto em 32 (37,6%) casos, em 36 (42,4%), a lesão foi considerada relacionada ao infarto e 17 (20,0%) pacientes não apresentavam lesões significativas nessa artéria, conforme descrito na Tabela 1 . Não houve diferença estatisticamente significativa entre as duas estratégias de revascularização em nenhuma das características analisadas, incluindo o tempo porta-balão entre os grupos.

**Tabela 1:** – Características clínicas e demográficas da população em estudo (n = 85)

		Estratégia de revascularização		
	Global	Completa	Incompleta	p
**Centro**				0,43
*1*	28 (32,9%)	17 (60,7%)	11 (39,3%)	
*2*	57 (67,1%)	41 (71,9%)	16 (28,1%)	
**Idade** (anos)	62±12	62,7±12	60,6±13	0,46
**Sexo masculino**	61 (71,8%)	42 (72,4%)	19 (74,0%)	0,99
**Raça branca**	80 (94,1%)	55 (94,8%)	25 (92,6%)	0,99
**História prévia**				
*HAS*	54 (63,5%)	37 (63,8%)	17 (63,0%)	0,99
*DM*	22 (25,9%)	14 (24,1%)	8 (29,6%)	0,79
*Tabagismo*	26 (30,6%)	21 (36,2%)	5 (18,5%)	0,16
*DAC prévia*	10 (11,8%)	5 (8,6%)	5 (18,5%)	0,34
**Localização do IAM**				0,94*
*Anterior*	37 (43,5%)	26 (44,8%)	11 (40,7%)	
*Inferior*	42 (49,4%)	28 (48,3%)	14 (51,9%)	
*Lateral*	6 (7,1%)	4 (6,9%)	2 (7,4%)	
**BRE**	4 (4,7%)	2 (3,4%)	2 (7,4%)	0,80*
**Nº de estenoses**				0,87
*2*	67 (78,8%)	46 (79,3%)	21 (77,8%)	
*3*	18 (21,1%)	12 (20,7%)	6 (22,2%)	
**ADA**				0,28
*Culpada*	32 (37,6%)	22 (37,9%)	10 (37,0%)	
*Não culpada*	36 (42,4%)	27 (46,6%)	9 (33,3%)	
*Sem lesão*	17 (20,0%)	9 (15,5%)	8 (29,6%)	
**FEVE < 50%**	41 (48,2%)	25 (43,1%)	16 (59,3%)	0,25
**Killip**				0,62*
*1*	71 (83,5%)	50 (86,2%)	21 (77,8%)	
*2*	7 (8,2%)	4 (6,9%)	3 (11,1%)	
*3*	7 (8,2%)	4 (6,9%)	3 (11,1%)	

Valores expressam média ± desvio padrão ou frequência absoluta e relativa. p: valor de probabilidade; para idade, análise de variância; demais, qui-quadrado de Pearson ou *teste exato de Fischer. HAS: hipertensão arterial sistêmica; DM: diabetes mellitus; DAC: doença arterial coronariana; IAM: infarto agudo do miocárdio; BRE: bloqueio de ramo esquerdo; ADA: artéria descendente anterior; FEVE: fração de ejeção do ventrículo esquerdo.

### Intervenção Coronariana

Dos 58 pacientes que receberam estratégia de RC, 6 (10,3%) realizaram tratamento completo no evento índice – todos apresentavam doença biarterial, sendo o tratamento da ANCI em 4 pacientes para o ramo diagonal e em 2 pacientes, para ADA. Os demais 52 pacientes realizaram o tratamento da ANCI de forma estadiada, sendo 38 na mesma internação e 14 em internação posterior. O tempo médio entre a ICP primária e o novo procedimento foi de 13±11 dias (variando entre 3 e 40 dias). Os detalhes referentes ao tratamento (ICP e terapia medicamentosa) estão descritos na Tabela 2 .

**Tabela 2:** – Intervenção coronariana e terapia medicamentosa (n = 85)

		Estratégia de revascularização		
	Global	Completa	Incompleta	p
**Trombolítico prévio**	3 (3,5%)	3 (5,2%)	0	0,57*
**Tipo de stent**				0,30*
*Convencional*	76 (89,4%)	50 (86,2%)	26 (96,3%)	
*Farmacológico*	9 (10,6%)	8 (13,8%)	1 (3,7%)	
**Inibidores da Glicoproteína IIb/IIIa**	23 (27,1%)	14 (24,1%)	9 (33,3%)	0,53
**Terapia clínica em 24h**				
*AAS*	85 (100%)	58 (100%)	27 (100%)	-
*Clopidogrel*	85 (100%)	58 (100%)	27 (100%)	-
*Estatina*	85 (100%)	58 (100%)	27 (100%)	-
*Betabloqueador*	43 (50,6%)	26 (44,8%)	17 (63,0%)	0,19
*IECA/BRA*	40 (47,1%)	27 (46,6%)	13 (48,1%)	0,99
*Nitrato*	30 (35,3%)	20 (34,5%)	10 (37,0%)	0,99
**Escore SYNTAX**				0,34*
*Baixo*	41 (48,2%)	30 (51,7%)	11 (40,7%)	
*Moderado*	44 (51,8%)	28 (48,3%)	16 (59,3%)	
*Alto*	—	—	—	

Valores expressam média ± desvio padrão ou frequência absoluta e relativa. p: valor de probabilidade; para idade, análise de variância; demais, qui-quadrado de Pearson ou *teste exato de Fischer. HAS: hipertensão arterial sistêmica; DM: diabetes mellitus; DAC: doença arterial coronariana; IAM: infarto agudo do miocárdio; BRE: bloqueio de ramo esquerdo; ADA: artéria descendente anterior; FEVE: fração de ejeção do ventrículo esquerdo.

Stents convencionais foram implantados em 76 (89,4%) pacientes. Todos receberam dupla antiagregação e estatina nas primeiras 24 horas. O uso de glicoproteína IIb/IIIa ocorreu em 23 (27,1%) casos. Com relação ao escore SYNTAX, 41 (48,2%) pacientes apresentaram escore baixo, enquanto 44 (51,8%) apresentaram escore moderado. Não houve casos com escore SYNTAX alto e não se observou diferença estatisticamente significativa entre as duas estratégias de revascularização quanto à distribuição do escore.

### Desfechos Clínicos

A mortalidade geral foi de 8,2%, sendo que 86% dos óbitos ocorreram no período intra-hospitalar. A chance de ocorrência tanto do desfecho primário quanto do secundário foi significativamente maior entre os indivíduos tratados com RI quando comparados com aqueles tratados com RC [razão de chances (OR) 5,1, intervalo de confiança de 95% (IC95%) 1,6-16,1 vs. OR 5,2, IC95% 1,2-22,9), respectivamente. Se analisada isoladamente a chance de óbito cardiovascular, o resultado foi semelhante (OR 6,4, IC95% 1,2-35,3), conforme descrito na Tabela 3 . Os óbitos ocorreram predominantemente no período intra-hospitalar, com apenas um paciente na estratégia de RI na fase tardia.

**Tabela 3:** – Desfechos clínicos e estratégia de revascularização (n = 85)

		Estratégia de revascularização		
	Completa n (%)	Incompleta n (%)	OR (IC95%)	p
**Desfecho primário (composto)**	6 (10,3%)	10 (37,0%)	5,10 (1,6-16,1)	0,005
*Óbito Cardiovascular*	2 (3,4%)	5 (18,5%)		
*Reinfarto*	—	—		
*Angina*	4 (6,9%)	5 (18,5%)		
**Desfecho secundário (composto)**	3 (5,17%)	6 (22,2%)	5,24 (1,2-22,9)	0,022
*AVE*	—	—		
*PCR não fatal*	2 (3,4%)	—		
*Sangramento maior*	1 (1,7%)	—		
*Reintervenção*	—	6 (22,2%)		

*OR: razão de chances; IC: intervalo de confiança; AVE: acidente vascular encefálico; PCR: parada cardiorrespiratória.*

**Figura 1:**
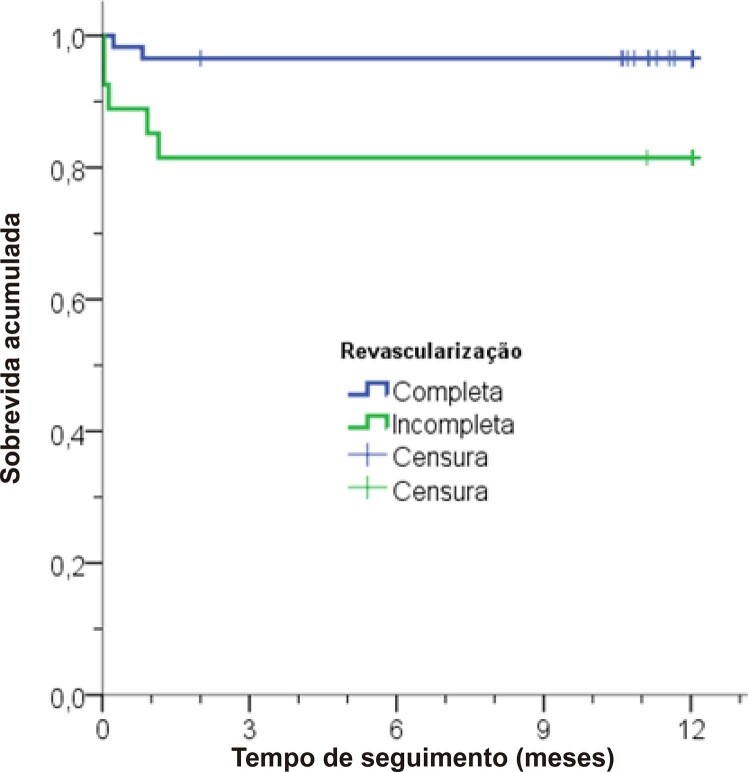
– Sobrevida após revascularização completa (RC) e incompleta (RI) para infarto agudo do miocárdio com supradesnivelamento do segmento ST (IAMCSST) com doença arterial coronariana (DAC) multiarterial em um período de 12 meses. *Teste de log-rank.

Conforme descrito na Tabela 4 , observou-se, na análise multivariável, que a RC estava associada a menor chance de ocorrência dos desfechos primário e secundário independente de sexo, idade, diabetes mellitus, lesão na descendente anterior como culpada pelo infarto, presença de lesão na descendente anterior e fração de ejeção < 50%. Ainda, a RC estava associada a menor chance de ocorrência do desfecho primário independente da parede ventricular acometida e da extensão da doença coronária. As curvas de Kaplan-Meier indicaram menor sobrevida em pacientes com doença coronariana multiarterial pós-IAMCSST submetidos a RI em um período de 12 meses (p = 0,017) ( Figura 1 ).

**Tabela 4:** – Associação independente entre estratégia estadiada e incidência de desfecho primário e secundário no seguimento de 1 ano (n = 85)

	Desfecho primário* OR (IC95%)	Desfecho secundário^†^ OR (IC95%)
**Não ajustado**	5,1 (1,6-16,1)	5,2 (1,2-22,9)
**Modelo 2** ^**‡**^	5,2 (1,6-16,5)	5,1 (1,1-23,0)
**Modelo 3** ^**§**^	5,1 (1,6-16,4)	4,9 (1,1-23,1)
**Modelo 4** ^**//**^	5,1 (1,6-16,4)	5,1 (1,1-24,1)
**Modelo 5** ^**¶**^	5,1 (1,6-16,7)	4,3 (0,9-21,0)
**Modelo 6** ^**#**^	4,6 (1,4-15,3)	3,6 (0,7-19,6)
Modelo 7**	4,7 (1,4-15,7)	2,3 (0,4-14,2)

OR: razão de chances; IC: intervalo de confiança. *Óbito, reinfarto, angina; ^†^Acidente vascular encefálico, parada cardiorrespiratória não fatal, sangramento, reintervenção. ^‡^ Ajustado para idade e sexo §Modelo 2 + ajuste para diabetes mellitus. //Modelo 3 + ajuste para artéria descendente anterior (ADA) culpada pelo evento. ^¶^Modelo 4 + lesão na ADA e fração de ejeção < 50%. #Modelo 5 + local do infarto. *Modelo 6 + número de lesões.

## Discussão

Em registro real da prática clínica, evidenciamos que a estratégia de RC está associada à redução significativa de desfechos duros no seguimento de 1 ano quando comparada à estratégia de RI e que o tratamento das ANCIs durante a intervenção coronária primária é incomum, sendo que a maioria dos pacientes com DAC multiarterial e IAMCSST receberam tratamento estadiado dentro de 40 dias do evento índice.

A presença de DAC multiarterial ocorre em aproximadamente 40-50% dos pacientes com IAMCSST^3 - 4^ e é considerada um poderoso preditor independente de mortalidade.^5^ Na nossa população em estudo, a prevalência foi de cerca de 35%. A história natural do IAMCSST demonstra que ocorrem distúrbios fisiopatológicos mais generalizados, com potencial de comprometer a perfusão coronariana além da distribuição da ACI, desestabilizando a placa ao longo do leito vascular coronariano.^13^ O processo patológico do IAMCSST envolve toda a árvore coronariana, sendo que a dinâmica desse processo inflamatório específico é maior no primeiro mês após o evento agudo,^14^ possivelmente explicando o aumento da taxa de mortalidade nos primeiros 30 dias,^15^ como visto no presente estudo. Devido ao pior prognóstico desses pacientes, quando se examina o papel da RC no contexto do IAMCSST, deve-se considerar o impacto dos fatores supracitados na determinação de como uma estratégia agressiva pode oferecer benefício clínico.^13^

Em consonância com as indicações das diretrizes contemporâneas, ainda conservadoras, na prática clínica do mundo real, vários registros demonstram que a abordagem de múltiplos vasos é adotada entre 9% e 24,4%.^16 - 18^ No registro português ProACS ( *Portuguese Registry of Acute Coronary Syndromes* ), por exemplo, esse número é de 19,2%. Neste estudo, a prática foi adotada em 68,2%. De acordo com alguns autores, a discrepância entre as diretrizes vigentes e a prática clínica resulta de diversos fatores, desde a falta de evidências clínicas até questões econômicas envolvendo fontes pagadoras e protocolos vigentes. O assunto continua a suscitar debates e só poderá ser resolvido com um estudo amplo e internacional.^19^ No contexto do tratamento multiarterial, também foi visto neste estudo a predominância de pacientes biarteriais (78,8%), em concordância com estudos como o PRAMI,^7^ e pacientes com menor complexidade de lesões – não houve casos de pacientes com escore SYNTAX alto –, o que nos leva à reflexão de que, provavelmente, os pacientes mais graves – triarteriais e com escore SYNTAX alto – receberam indicação de tratamento cirúrgico após a angioplastia primária nos centros estudados.

Com relação à terapia farmacológica, seguindo as diretrizes para IAMCSST, 100% dos pacientes receberam dupla antiagregação plaquetária e estatinas. O uso de inibidores da glicoproteína IIb/IIIa não diferiu entre os grupos, ficando em torno de 27,1% dos casos, embora tenha sido amplamente avaliado por metanálise que concluiu que seus benefícios são maiores em pacientes de alto risco, como naqueles submetidos à RC.^20^ Outro fator de destaque é a alta taxa de uso de stent convencional – 89,4% dos casos –, o que difere dos ensaios clínicos randomizados (ECRs) a respeito deste tema.^7 - 10^ Tal fato chama a atenção para a disparidade entre os pacientes incluídos em ECRs e os pacientes reais, o que reforça a importância de registros populacionais como esses. Embora os ECRs empreguem o delineamento mais amplamente aceito para comparar tratamentos, eles ainda têm deixado muitas questões importantes sem resposta. Acredita-se que uma análise cuidadosa das informações de registros clínicos ofereça uma abordagem complementar aos ensaios clínicos, especialmente considerando a potencial inclusão de amostras mais representativas da população-alvo. Além disso, devido ao fato de os ECRs serem realizados em centros de excelência, não fica claro se seus resultados podem ser generalizados para a prática clínica usual. A experiência do operador, por exemplo, varia entre as instituições e pode interferir no resultado. Através de registros como o presente estudo, podemos perceber que, mesmo em condições não ideais, permanece significativo o benefício da RC em pacientes multiarteriais.^21^ Entre 2006 e 2010, o registro SCAAR ( *Swedish Coronary Angiography and Angioplasty Registry* ) acompanhou a evolução de 23.342 pacientes com doença de múltiplos vasos que realizaram angioplastia coronária com RI e sua possível associação com morte, nova intervenção e infarto agudo do miocárdio a longo prazo. A RI no momento da alta hospitalar se associou a um alto risco de eventos cardíacos adversos em um ano, com um risco ajustado de morte e a combinação de morte/infarto de 1,29 (IC95% 1,12-1,49; p = 0,0005) e 1,42 (IC95% 1,30-1,56; p < 0,0001), respectivamente.

O principal achado deste estudo é a demonstração de significativo benefício na redução da mortalidade em pacientes submetidos à RC, mesmo quando realizada de forma estadiada. Os casos de RC no evento índice foram incomuns, realizados somente em pacientes biarteriais, com anatomia favorável e menor gravidade na chegada (Killip I). Outro achado importante foi o significativo benefício com relação a revascularização repetida e angina recorrente. Em pacientes com IAMCSST tratados com ICP primária em hospitais reais, a RC não aumenta a mortalidade em curto e longo prazos, mostrando-se segura quando realizada de forma estadiada.^22^

Embora o benefício da estratégia estadiada após a ICP primária tenha sido sugerido em diversos estudos, como neste, permanecem questionamentos a serem resolvidos, como o tempo apropriado para a ICP estadiada. Na prática clínica, fatores como disfunção renal, complexidade das lesões, volume de contraste, dose de radiação, estado hemodinâmico e *status* do paciente podem influenciar a decisão pelo tempo ideal da revascularização. Uma pesquisa via eletrônica conduzida pela ACC revelou que, embora a maioria dos cardiologistas intervencionistas concorde em realizar a RC de forma estadiada, houve variabilidade significativa nas opiniões no que se refere ao tempo ideal para a próxima ICP. Somente 22% dos entrevistados realizavam a nova intervenção na mesma hospitalização; a maioria recomendou um prazo acima de 15 dias para o segundo procedimento.^23^

Apesar das evidências e estudos em andamento, nenhum estudo pode ser capaz de definir uma estratégia única para pacientes com IAMCSST e DAC multiarterial. Como são pacientes heterogêneos, a estratégia deve ser individualizada. Desnecessário dizer que o foco deve ser no tratamento da lesão culpada. A decisão deve levar em conta a complexidade anatômica, função ventricular e perfil do paciente, a fim de se buscar a melhor estratégia, idealmente através de um *heart team* . Uma estratificação de risco completa, com dados clínicos e angiográficos, é crucial para a melhor avaliação destes pacientes.^24^

O presente estudo apresenta algumas limitações que devem ser consideradas, especialmente relacionadas ao seu caráter observacional. Não se pode excluir a possibilidade de viés de seleção, apesar de não se ter identificado diferenças estatisticamente significativas quanto às variáveis avaliadas em relação às características basais dos pacientes tratados com RC ou RI e de não se ter observado modificação do efeito da estratégia sobre a ocorrência de desfecho primário pelos fatores considerados na análise multivariável, uma vez que a estratégia de intervenção ficou a critério do operador. Além disso, trata-se de um estudo com número pequeno de pacientes de dois centros do Sul do Brasil, podendo não ser representativo de realidades de outras regiões e serviços não públicos.

## Conclusões

No presente estudo, com dados reais da prática clínica de dois centros do Sul do Brasil, encontramos que, em pacientes com DAC multiarterial no contexto do IAMCSST e submetidos à ICP primária, a estratégia de RC está associada com redução significativa dos desfechos primário e secundário no seguimento de 1 ano, quando comparada à RI. Esses dados devem suscitar discussão sobre os protocolos clínicos e institucionais vigentes.

## References

[1] 1. Sekercioglu N, Spencer FA, Lopes LC, Guyatt GH. Culprit vessel only vs immediate complete revascularization in patients with acute ST-Segment elevation myocardial infarction: systematic review and meta-analysis. Clin Cardiol. 2014;37(12):765-72. 10.1002/clc.22333 PMC6647587 25236941

[2] 2. Zhang D, Song X, Lv S, Yuan F, Xu F, Zhang M, et al. Culprit vessel only versus multivessel percutaneous coronary intervention in patients presenting with ST-segment elevation myocardial infarction and multivessel disease. PLoS One. 2014;9(3):e92316. 10.1371/journal.pone.0092316 PMC3961318 24651489

[3] 3. Park DW, Clare RM, Schulte PJ, Pieper KS, Shaw LK, Califf RM, et al. Extent, location, and clinical significance of non-infarct-related coronary artery disease among patients with ST-elevation myocardial infarction. JAMA. 2014;312(19):2019-27. 10.1001/jama.2014.15095 25399277

[4] 4. Levine GM, Bates ER, Balnkeship JC, Bailey SR, Bitti JA, Cercek J, et al. 2015 ACC/AHA/SCAI Focused Update on Primary Percutaneous Coronary Intervention for Patients With ST-Elevation Myocardial Infarction: An Update of the 2011 ACCF/AHA/SCAI Guideline for Percutaneous Coronary Intervention and the 2013 ACCF/AHA Guideline for the Management of ST-Elevation Myocardial Infarction. A Report of the American College of Cardiology/American Heart Association Task Force on Clinical Practice Guidelines and the Society for Cardiovascular Angiography and Interventions. Circulation. 2016;133:1135-47. 10.1161/CIR.0000000000000336 26490017

[5] 5. Wolny R, Pregowski J, Bekta P, Chmielak Z, Witkowski A. Early occlusion of the non-infarct-related coronary artery following successful primary percutaneous coronary intervention in ST-elevation myocardial infarction. Postepy Kardiol Interwencyjnej. 2015;11(2):136-40. 10.5114/pwki.2015.52287 PMC4495130 26161106

[6] 6. Levine GM, Bates ER, Blankenship JC, Bailey SR, Bitti JA, Cercek J, et al. 2011 ACCF/AHA/ SCAI guideline for percutaneous coronary intervention: a report of the American College of Cardiology Foundation/American Heart Association Task Force on Practice Guidelines and the Society for Cardiovascular Angiography and Interventions. Circulation. 2011;124:e574–651. 10.1161/CIR.0b013e31823ba622 22064601

[7] 7. Wald DS, Morris JK, Wald NJ, Chase AJ, Edwards RJ, Hughes LO, et al. Randomized trial of preventive angioplasty in myocardial infarction. N Engl J Med. 2013;369(12):1115-23. 10.1056/NEJMoa1305520 23991625

[8] 8. Kelly DJ, Mc Cann GP, Blackman D, Curzen NP, Dalby M, Grennwood JP, et al. Randomized trial of complete versus lesion-only revascularization in patients undergoing primary percutaneous coronary intervention for STEMI and multivessel disease: the CvLPRIT trial. J Am Coll Cardiol. 2015;65(10):963-72. 10.1016/j.jacc.2014.12.038 PMC4359051 25766941

[9] 9. Hlinomaz O. Multivessel coronary disease diagnosed at the time of primary PCI for STEMI: complete revascularization versus conservative strategy. PRAGUE 13 trial. [citado 10 Set. 2015]. Disponível em: http://sbhci.org.br/wp-content/uploads/2015/05/PRAGUE-13-Trial.pdf.

[10] 10. Engstrøm T, Kelbæk H, Helqvist S, Hofsten DE, Klovgaard L, Holmvang L, et al. Complete revascularization versus treatment of the culprit lesion only in patients with ST-segment elevation myocardial infarction and multivessel disease (DANAMI 3-PRIMULTI): an open-label, randomized controlled trial. Lancet. 2015;386(9994):665-71. 10.1016/s0140-6736(15)60648-1 26347918

[11] 11. Ibanes B, James S, Agewall S, Antunes MJ, Bucciarelli-Ducci C, Bueno H, et al. 2017 ESC Guidelines for the management of acute myocardial infarction in patients presenting with ST-segment elevation: The Task Force for the management of acute myocardial infarction in patients presenting with ST-segment elevation of the European Society of Cardiology (ESC). Eur Heart J. 2018;39(2):119-77. 10.1093/eurheartj/ehx393 28886621

[12] 12. Piegas LS, Timerman A, Feitosa GS, Nicolau JC, Mattos LAP, Andrade MD, et al. V Diretriz da Sociedade Brasileira de Cardiologia sobre Tratamento do Infarto Agudo do Miocárdio com Supradesnível do Segmento ST. Arq Bras Cardiol. 2015;105(2 Suppl 1):1-105. 10.5935/abc.20150107 26375058

[13] 13. Pollack A, Mohanty BD, Handa R, Looser PM, Fuster V, King III Sb, et al. Preventive stenting in acute myocardial infarction. JACC Cardiovasc Interv. 2015;8(1 PtB):131-8. 10.1016/j.jcin.2014.09.006 25616917

[14] 14. Goldstein JA, Demetriou D, Grines CL, Pica M, Shoukfeh M, O´Neill WW. Multiple complex coronary plaques in patients with acute myocardial infarction. N Engl J Med. 2000;343(13):915-22. 10.1056/NEJM200009283431303 11006367

[15] 15. Lekston A, Tajstra M, Gasior M, Gierlotka M, Pres D, Hudzik B, et al. Impact of multivessel coronary disease on one-year clinical outcomes and five-year mortality in patients with ST-elevation myocardial infarction undergoing percutaneous coronary intervention. Kardio Pol. 2011;69(4):336-43. 21523666

[16] 16. Dziewierz A, Siudak Z, Rakowski T, Zasada W, Dubiel JS, Dudek D. Impact of multivessel coronary artery disease and noninfarct-related artery revascularization on outcome of patients with ST-elevation myocardial infarction transferred for primary percutaneous coronary intervention (from the EUROTRANSFER Registry). Am J Cardiol. 2010;106(3):342-7. 10.1016/j.amjcard.2010.03.029 20643243

[17] 17. Bengalore S, Kumar S, Poddar KL, Ramasamy S, Rha SW, Faxon DP. Meta-analysis of multivessel coronary artery revascularization versus culprit only revascularization in patients with ST-segment elevation myocardial infarction and multivessel disease. Am J Cardiol. 2011;107(9):1300-10. 10.1016/j.amjcard.2010.12.039 21349487

[18] 18. Kornowski R, Mehran R, Dangas G, Nikolsky E, Assali A, Claessen BE, et al. Prognostic impact of staged versus “one-time” multivessel percutaneous intervention in acute myocardial infarction: analysis from the HORIZONS-AMI (harmonizing outcomes with revascularization and stents in acute myocardial infarction) trial. J Am Coll Cardiol. 2011;58(7):704-11. 10.1016/j.jacc.2011.02.071 21816305

[19] 19. Santos AR, Piçarra BC, Celeiro M, Bento A, Aguiar J. Multivessel approach in ST-elevation myocardial infarction: impact on in-hospital morbidity and mortality. Rev Port Cardiol. 2014;33(2):67-73. 10.1016/j.repc.2013.07.015 24502933

[20] 20. De Luca G, Navarese E, Marino P. Risk profile and benefits from Gp IIb-IIIa inhibitors among patients with ST-segment elevation myocardial infarction treated with primary angioplasty: a meta-regression analysis of randomized trials. Eur Heart J. 2009;30(22):2705-13. 10.1093/eurheartj/ehp118 PMC2777025 19875386

[21] 21. Califf RM, Pryor DB, Greenfield JC. Beyond randomized trials: applying clinical experience in the treatment of patients with coronary artery disease. Circulation. 1986;74(6):1191-94. 10.1161/01.cir.74.6.1191 3536148

[22] 22. Jensen LO, Thayssen P, Farkas DK, Hougaard M, Tekelsen CJ, Tilsted HH, et al. Culprit only or multivessel percutaneous coronary interventions in patients with ST-segment elevation myocardial infarction and multivessel disease. EuroIntervention. 2012;8(4):456-64. 10.4244/EIJV8I4A72 22917729

[23] 23. Hambraeus K, Jensevik K, Lagerqvist B, Lindahl B, Carlsson R, Farzaneh-Far R, et al. Long-Term Outcome of Incomplete Revascularization After Percutaneous Coronary Intervention in SCAAR (Swedish Coronary Angiography and Angioplasty Registry. JACC Cardiovasc Interv. 2016;9(3):207-15. 10.1016/j.jcin.2015.10.034 26847112

[24] 24. Cuisset T, Noc M. Multivessel PCI in STEMI: ready to be the recommended strategy? EuroIntervention. 2014;10(Suppl T):T47-54. 10.4244/EIJV10STA9 25256534

