# Supplementary material for: Revascularização Completa Versus Tratamento da Artéria Culpada no Infarto com Supradesnivelamento do Segmento ST: Registro Multicêntrico
Source: Arq Bras Cardiol. 2020 Aug 19;115(2):229–37. [Article in Portuguese] doi: 10.36660/abc.20180346 (PMC8384275; doi:10.36660/abc.20180346)
Supplement: Translation

# Translation: Complete Revascularization Versus Treatment of the Culprit Artery Only in ST Elevation Myocardial Infarction: A Multicenter Registry

**Authors:** Julia Cremona Cadore, Mariana Vargas Furtado, Rogério Tumelero, Alexandre Tognon, Ana Maria Krepsky, Denis Cadore, Karen Brasil Ruschel, Julia Caldas Bedin, Thais Conte, Carisi Anne Polanczyk

**Affiliations:** 1 Brazil Universidade Federal do Rio Grande do Sul - PPG Ciências Cardiovasculares: Cardiologia, Porto Alegre, RS - Brazil; 2 Brazil Hospital de Clínicas de Porto Alegre - Instituto de Avaliação de Tecnologia em Saúde, Porto Alegre, RS - Brazil; 3 Brazil Associação Hospitalar Beneficente São Vicente de Paulo, Passo Fundo, RS - Brasil; 4 Brazil Universidade Federal do Rio Grande do Sul - Faculdade de Medicina, Porto Alegre, RS - Brazil; 5 Brazil Hospital de Clínicas de Porto Alegre, Porto Alegre, RS – Brazil

**Keywords:** ST Elevation Myocardial Infarction/mortality, Cohort Studies, Hemodynamic, Death Certificates, Angina Pectoris, Stroke, Heart Arrest, Angina Pectoris, Stroke, Heart Arrest, Percutaneous Coronary Interventions

## Abstract

**Background:**

Data on the management and prognosis of patients with ST-segment elevation myocardial infarction (STEMI) and multivessel disease are limited in Brazil, showing that the available revascularization strategies should be investigated

**Objective:**

To assess the outcomes of complete revascularization versus treatment of the culprit artery only in patients with STEMI and multivessel disease.

**Methods:**

A prospective cohort study was conducted at two medical centers in southern Brazil with a 1-year follow-up after the index procedure. The primary outcome was a composite of cardiac death, reinfarction, or recurrent angina, while the secondary outcome was stroke, nonfatal cardiac arrest, major bleeding, or need for reintervention. The probability of outcomes occurring was compared between the groups using binary logistic regression. A p-value < 0.05 was considered statistically significant.

**Results:**

Eighty-five patients were included. Their mean age was 62±12 years, and 61 (71.8%) were male. Fifty-eight (68.2%) were treated with complete revascularization and 27 (31.8%) with incomplete revascularization. The chance of both the primary and secondary outcomes occurring was significantly greater among patients treated with incomplete revascularization when compared to those treated with complete revascularization (odds ratio [OR] 5.1, 95% confidence interval [CI] 1.6-16.1 vs. OR 5.2, 95% CI 1.2-22.9, respectively), as well as cardiac death (OR 6.4, 95% CI 1.2-35.3).

**Conclusion:**

Registry data from two centers in southern Brazil demonstrate that the complete revascularization strategy is associated with a significant reduction in primary and secondary outcomes in a 1-year follow-up when compared to the incomplete revascularization strategy (Arq Bras Cardiol. 2020; 115(2):229-237)

## Introduction

ST-segment elevation myocardial infarction (STEMI) is an extremely relevant public health issue^1^ with a high mortality rate if not properly treated.^2^ Approximately 50% of patients present with multivessel coronary artery disease (CAD),^3 - 4^ in which case prognosis is even more unfavorable.^5^

Therapeutic options for this complex group of patients with STEMI and multivessel disease include primary percutaneous coronary intervention (PCI) in the culprit artery and PCI in the other stenoses only for spontaneous ischemia or risk findings in noninvasive tests (incomplete revascularization – IR); multivessel PCI at the time of primary PCI (complete revascularization – CR); primary PCI in the culprit artery and staged approach of the other stenoses (staged CR). Initial studies showed conflicting results.^6^ The PRAMI (Preventive Angioplasty in Acute Myocardial Infarction) study, however, led to a paradigm shift as it demonstrated the benefit of multivessel PCI compared to culprit-artery-only PCI.^7^ Other trials reinforced the hypothesis that a CR strategy could be beneficial and safe in selected patients with STEMI.^8 - 10^

Based on those findings, the American College of Cardiology (ACC) and the American Heart Association (AHA) updated their guidelines in 2015 with recommendations for both CR and staged approach at the time of primary PCI in hemodynamically stable patients.^4^ The 2017 European Society of Cardiology (ESC) guideline followed the same path.^11^ The 2015 Brazilian Society of Cardiology (SBC) guideline, in turn, finds it reasonable to treat less complex, severe stenosis located in the coronary system linked to the infarct-related vessel.^12^ Conversely, the guideline highlights that these patients are more likely to experience new coronary events within 1 year, thus suggesting that severe coronary stenoses that are not directly related to the index procedure should be managed later with a staged approach.^12^

In this study, we aimed to assess real-life outcomes of CR versus treatment of the culprit artery only in patients with STEMI and multivessel disease managed at two hospitals in southern Brazil.

## Method

### Study Design

A registry study was conducted to evaluate patients with STEMI and multivessel CAD admitted to two medical centers in southern Brazil. Prospective data were collected from October 2015 to March 2016 using hospital admission information. Also, retrospective data were collected from January to September 2015 by reviewing medical records. Primary and secondary outcomes were prospectively assessed by monthly telephone contact for 12 months following hospital discharge for the index event.

### Patient Selection

Male and female patients were included if they were aged ≥ 18 years, were admitted to the study centers in the 6-month period, had diagnosis of STEMI treated with primary PCI, and presented with multivessel CAD on coronary angiography – defined as the presence of a lesion ≥ 70% by visual assessment of the angiogram in at least two projections in more than one coronary artery. Patients who were referred to the hospitals for rescue angioplasty after thrombolytic therapy and had multivessel CAD were also eligible.

Patients were excluded if they had undergone previous coronary artery bypass grafting (CABG), had cardiogenic shock at admission, indication for CABG following primary angioplasty, left main coronary artery disease, lesion in the proximal portion of the left anterior descending artery (LAD) or the circumflex artery, or chronic total occlusion of a nonculprit artery (cases which would benefit from CABG at the discretion of the health care team).

### Data Collection

Study data were collected using a standardized form for the period of hospitalization for treatment of the acute event, including demographic characteristics, tests performed in the emergency department, coronary angiography results, administered treatment, as well as follow-up data for a 1-year period. All procedures related to patient care were the responsibility of the health care team, with no influence from the researchers. The study was conducted in accordance with Brazilian resolution no. 466/2012 and was approved by the Research Ethics Committees at both institutions. The prospectively enrolled patients signed an informed consent form after the initial test (coronary angiography); for retrospective data collection, the researchers signed a data confidentiality agreement.

### Follow-up and Outcomes of Interest

Progression and occurrence of in-hospital outcomes were assessed during hospitalization and, subsequently, via telephone contact and review of medical records. The primary outcome was defined as the occurrence of: (1) death from cardiovascular causes; (2) reinfarction, defined as recurrent ischemic pain (although not mandatory), new ST-segment elevation ≥ 0.1 mV or new Q wave in at least two contiguous leads or abnormal (above the upper limit of normal according to the reference range used by the local laboratory or at least 50% above the value in the previous test) levels of serum markers (troponin or creatine kinase-MB); or (3) recurrent angina, defined as persistent pain, need for sublingual nitrate, or readmission due to recurrent angina.

The secondary outcome was a composite of: (1) stroke; (2) nonfatal cardiac arrest; (3) major bleeding (defined as the need for blood transfusion due to a drop of more than 3 g/dL in the hemoglobin test, and/or hemoglobin level below 10 g/dL, and/or hemodynamic instability, and/or prolonged hospitalization due to major bleeding, and/or hemorrhagic stroke; or (4) need for unplanned percutaneous or surgical reintervention.

### Statistical Analysis

Statistical analysis was performed using IBM SPSS Statistics version 22.0 for Windows. Age and time between primary PCI and new procedure were described as mean ± standard deviation. Categorical variables were described as absolute and relative frequency. The distributions of numerical variables were compared between the CR and culprit-artery-only groups using analysis of variance with a single criterion of classification, while those of categorical variables were compared using Pearson’s chi-square test with continuity correction or Fisher’s exact test whenever appropriate. The chance of primary outcome, secondary outcome, and death from all causes occurring were compared between the groups described above using binary logistic regression. In multivariable analysis, models were compared using the likelihood ratio test. Kaplan-Meier curves for STEMI were calculated for CR and IR. Differences in survival rate were assessed by the log-rank test. Odds ratios were described with their respective 95% confidence intervals. Tests with p-value < 0.05 were considered statistically significant. (Seria cardiovascular causes?)

## Results

From January 2015 to March 2016, 248 patients with a diagnosis of STEMI were admitted to the emergency departments at the two study centers; of those, 85 (34.3%) patients had multivessel CAD.

Of the total 85 patients, 58 (68.2%) were treated with CR and 27 (31.8%) were treated with culprit-artery-only revascularization. The mean age was 62±12 years, and 61 (71.8%) participants were male. Inferior infarction occurred in 42 (49.4%) patients, followed by anterior infarction in 37 (43.5%). Seventy-one (83.5%) patients were rated as Killip class I at admission, and 68 (78.8%) had double-vessel disease. The LAD was responsible for 32 (37.6%) infarctions, while the lesion was found to be related to the acute myocardial infarction in 36 (42.4%) patients. Finally, 17 (20.0%) patients had no significant lesions in that artery, as described in Table 1 . There was no statistically significant difference between the two revascularization strategies in any of the characteristics that were analyzed, including door-to-balloon time.

**Table 1:** – Clinical and demographic characteristics of the study population (n = 85)

		Revascularization strategy		
	Overall	Complete	Incomplete	p
**Center**				0.43
*1*	28 (32.9%)	17 (60.7%)	11 (39.3%)	
*2*	57 (67.1%)	41 (71.9%)	16 (28.1%)	
**Age** (years)	62±12	62.7±12	60.6±13	0.46
**Male**	61 (71.8%)	42 (72.4%)	19 (74.0%)	0.99
**White**	80 (94.1%)	55 (94.8%)	25 (92.6%)	0.99
**Previous history**				
*Hypertension*	54 (63.5%)	37 (63.8%)	17 (63.0%)	0.99
*Diabetes*	22 (25.9%)	14 (24.1%)	8 (29.6%)	0.79
*Smoking*	26 (30.6%)	21 (36.2%)	5 (18.5%)	0.16
*Previous CAD*	10 (11.8%)	5 (8.6%)	5 (18.5%)	0.34
**AMI location**				0.94*
*Anterior*	37 (43.5%)	26 (44.8%)	11 (40.7%)	
*Inferior*	42 (49.4%)	28 (48.3%)	14 (51.9%)	
*Lateral*	6 (7.1%)	4 (6.9%)	2 (7.4%)	
**LBBB**	4 (4.7%)	2 (3.4%)	2 (7.4%)	0.80*
**No. of stenoses**				0.87
*2*	67 (78.8%)	46 (79.3%)	21 (77.8%)	
*3*	18 (21.1%)	12 (20.7%)	6 (22.2%)	
**LAD**				0.28
*Culprit*	32 (37.6%)	22 (37.9%)	10 (37.0%)	
*Nonculprit*	36 (42.4%)	27 (46.6%)	9 (33.3%)	
*No lesion*	17 (20.0%)	9 (15.5%)	8 (29.6%)	
**LVEF < 50%**	41 (48.2%)	25 (43.1%)	16 (59.3%)	0.25
**Killip class**				0.62*
*1*	71 (83.5%)	50 (86.2%)	21 (77.8%)	
*2*	7 (8.2%)	4 (6.9%)	3 (11.1%)	
*3*	7 (8.2%)	4 (6.9%)	3 (11.1%)	

Values are mean ± standard deviation or absolute and relative frequency. p: probability value; analysis of variance was used for age; for the others, Pearson’s chi-square text or *Fischer’s exact test was used. CAD: coronary artery disease; AMI: acute myocardial infarction; LBBB: left bundle branch block; LAD: left anterior descending artery; LVEF: left ventricular ejection fraction.

### Coronary Intervention

Of the 58 patients who were given the CR strategy, 6 (10.3%) were fully treated at the index event – all of them had double-vessel disease, including four patients with the diagonal branch and two patients with the LAD as the nonculprit artery that was treated. Fifty-two patients were treated with staged revascularization of the nonculprit artery – 38 at the initial admission and 14 at a subsequent admission. The mean time between primary PCI and the new procedure was 13±11 days, ranging from 3 to 40 days. Detailed treatment (including PCI and drug therapy) is described in Table 2 .

**Table 2:** – Coronary intervention and drug therapy (n = 85)

		Revascularization strategy		
	Overall	Complete	Incomplete	p
**Previous thrombolytic therapy**	3 (3.5%)	3 (5.2%)	0	0.57*
**Stent type**				0.30*
*Bare-metal*	76 (89.4%)	50 (86.2%)	26 (96.3%)	
*Drug-eluting*	9 (10.6%)	8 (13.8%)	1 (3.7%)	
**Glycoprotein IIb/IIIa inhibitors**	23 (27.1%)	14 (24.1%)	9 (33.3%)	0.53
**Medical therapy within 24 h**				
*ASA*	85 (100%)	58 (100%)	27 (100%)	-
*Clopidogrel*	85 (100%)	58 (100%)	27 (100%)	-
*Statin*	85 (100%)	58 (100%)	27 (100%)	-
*Beta-blocker*	43 (50.6%)	26 (44.8%)	17 (63.0%)	0.19
*ACEI/ARB*	40 (47.1%)	27 (46.6%)	13 (48.1%)	0.99
*Nitrate*	30 (35.3%)	20 (34.5%)	10 (37.0%)	0.99
**SYNTAX score**				0.34*
*Low*	41 (48.2%)	30 (51.7%)	11 (40.7%)	
*Moderate*	44 (51.8%)	28 (48.3%)	16 (59.3%)	
*High*	—	—	—	

Values are absolute and relative frequency. p: probability value; analysis of variance was used for age; for the others, Pearson’s chi-square text or *Fischer’s exact test was used. ASA: acetylsalicylic acid; ACEI: angiotensin-converting enzyme inhibitor; ARB: angiotensin receptor blocker.

Bare-metal stents were implanted in 76 (89.4%) patients. All patients were given dual antiplatelet therapy and statin within 24 hours. Glycoprotein IIb/IIIa inhibitor was used in 23 (27.1%) patients. Regarding the SYNTAX score, 41 (48.2%) patients had a low score, while 44 (51.8%) had a moderate score. No patient had a high SYNTAX score, and there was no statistically significant difference between the two revascularization strategies regarding the distribution of the scores.

### Clinical Outcomes

Overall mortality was 8.2%, and 86% of deaths occurred in hospital. The chance of both the primary and secondary outcomes occurring was significantly greater among individuals treated with IR when compared to those treated with CR (OR 5.1, 95% CI 1.6-16.1 vs. OR 5.2, 95% CI 1.2-22.9, respectively). If the chance of cardiac death is analyzed separately, the result was similar (OR 6.4, 95% CI 1.2-35.3), as described in Table 3 . Deaths occurred predominantly in hospital, and only one patient died in the late stage of the IR strategy.

**Table 3:** – Clinical outcomes according to revascularization strategy (n = 85)

		Revascularization strategy		
	Complete n (%)	Incomplete n (%)	OR (95% CI)	p
**Primary outcome (composite)**	6 (10.3%)	10 (37.0%)	5.10 (1.6-16.1)	0.005
*Cardiac death*	2 (3.4%)	5 (18,5%)		
*Reinfarction*	—	—		
*Angina*	4 (6.9%)	5 (18.5%)		
**Secondary outcome (composite)**	3 (5.17%)	6 (22.2%)	5.24 (1.2-22.9)	0.022
*Stroke*	—	—		
*Nonfatal cardiac arrest*	2 (3.4%)	—		
*Major bleeding*	1 (1.7%)	—		
*Reintervention*	—	6 (22.2%)		

*OR: odds ratio; CI: confidence interval.*

In multivariate analysis, as described in Table 4 , CR was associated with a decreased chance of both primary and secondary outcomes occurring regardless of sex, age, diabetes, culprit lesion in the LAD, and presence of LAD lesion and ejection fraction < 50%. Also, CR was associated with a decreased chance of the primary outcome occurring regardless of the ventricular wall affected and the extent of CAD. The Kaplan-Meier curves showed a decreased 12-month survival in patients with multivessel disease post-STEMI who underwent IR (p = 0.017) ( Figure 1 ).

**Table 4:** – Independent association between staged revascularization strategy and incidence of primary and secondary outcomes in a 1-year follow-up (n = 85)

	Primary outcome* OR (95% CI)	Secondary outcome^†^OR (95% CI)
**Unadjusted**	5.1 (1.6-16.1)	5.2 (1.2-22.9)
**Model 2** ^**‡**^	5.2 (1.6-16.5)	5.1 (1.1-23.0)
**Model 3** ^**§**^	5.1 (1.6-16.4)	4.9 (1.1-23.1)
**Model 4** ^**//**^	5.1 (1.6-16.4)	5.1 (1.1-24.1)
**Model 5** ^**¶**^	5.1 (1.6-16.7)	4.3 (0.9-21.0)
**Model 6** ^**#**^	4.6 (1.4-15.3)	3.6 (0.7-19.6)
Model 7**	4.7 (1.4-15.7)	2.3 (0.4-14.2)

OR: odds ratio; CI: confidence interval. *Death, reinfarction, angina;^†^Stroke, nonfatal cardiac arrest, major bleeding, reintervention.^‡^Adjusted for age and sex.^§^Model 2 + adjustment for diabetes.^//^Model 3 + adjustment for culprit lesion in left anterior descending artery (LAD).^¶^Model 4 + LAD lesion and ejection fraction < 50%.^#^Model 5 + infarction location. **Model 6 + number of lesions.

**Figure 1:** – Twelve-month survival after complete revascularization (CR) and incomplete revascularization (IR) in patients with acute ST-segment elevation myocardial infarction (STEMI) and multivessel disease. *Log-rank test.

## Discussion

In this real-world practice registry, we showed that the CR strategy is associated with a significant reduction in hard outcomes in a 1-year follow-up when compared to the IR strategy. Also, the treatment of nonculprit arteries during primary PCI was uncommon, as most patients with multivessel CAD and STEMI were managed with a staged approach within 40 days of the index event.

Multivessel CAD occurs in approximately 40-50% of patients with STEMI^3 - 4^ and is considered a strong independent predictor of mortality.^5^ In our study population, the prevalence was about 35%. The natural history of STEMI demonstrates that the occurrence of more generalized pathophysiological derangements has the potential to compromise coronary perfusion beyond the culprit artery distribution and destabilize plaque throughout the coronary vascular bed.^13^ The pathological process of STEMI involves the entire coronary tree, and the dynamics of this specific inflammatory process is greater in the first month following the acute event,^14^ which may explain an increased mortality rate within 30 days,^15^ as seen in the present study. Because these patients have poor prognosis, the role of CR within the context of STEMI should be examined considering the impact of the aforementioned factors on determining whether an aggressive strategy could provide clinical benefit.^13^

Consistent with the still conservative recommendations of contemporary guidelines for real-world clinical practice, several registries demonstrate that the use of multivessel approach ranges from 9% to 24.4%.^16 - 18^ In the ProACS (Portuguese Registry of Acute Coronary Syndromes) registry, for example, the rate was 19.2%. In our study, the approach was used in 68.2%. According to some authors, discrepancy between current guidelines and clinical practice results from several factors, including lack of clinical evidence and economic issues involving paying sources and current protocols. The subject is controversial and will only be resolved with a broad international study.^19^ Within the context of multivessel treatment, our study showed a predominance of patients with double-vessel disease (78.8%), in agreement with studies such as the PRAMI trial,^7^ and patients with less complex lesions, as there were no participants with a high SYNTAX score. Those findings suggest that more severe patients – with triple-vessel disease and a high SYNTAX score – had indication for surgical treatment following primary angioplasty at the study centers.

With regard to drug therapy, all patients were given dual antiplatelet therapy and statins as recommended in STEMI guidelines. There was no difference in the use of glycoprotein IIb/IIIa inhibitors between the groups (around 27.1%), although a large metanalysis concluded that greater benefits are observed in high-risk patients, such as those undergoing CR.^20^ Another highlight is the high rate of bare-metal stenting (89.4%), which differs from the results of randomized clinical trials (RCTs).^7 - 10^ This shows a disparity between patients included in RCTs and real-world patients, which reinforces the importance of population registries. Although RCTs use the most widely accepted design for comparing treatments, they have left many important questions unanswered. Careful review of clinical registry information is believed to provide a complementary approach to RCTs, especially because of the potential inclusion of more representative samples of the target population. Furthermore, as RCTs are conducted at centers of excellence, it remains unclear whether their results can be generalized to usual clinical practice. The operator’s experience, for example, varies across institutions and may interfere with the results. Registries such as the present study show that even in suboptimal conditions the benefit of CR in patients with multivessel disease remains significant.^21^ The SCAAR (Swedish Coronary Angiography and Angioplasty Registry) study followed up from 2006 to 2010 a total of 23,342 patients with multivessel disease who underwent coronary angioplasty with IR and assessed its long-term association with death, new intervention, and myocardial infarction. IR at the time of hospital discharge was associated with a high risk of adverse cardiac events within 1 year, with an adjusted hazard ratio of death and the combination of death/infarction of 1.29 (95% CI 1.12-1.49; p = 0.0005) and 1.42 (95% CI 1.30-1.56; p < 0.0001), respectively.

This study was able to demonstrate a significant benefit of CR in reducing mortality, even when a staged approach was used. CR at the index event was uncommon, being performed only in patients with double-vessel disease, favorable anatomy, and lower severity at admission (Killip class I). Another important finding was the significant benefit regarding repeat revascularization and recurrent angina. In patients with STEMI treated with primary PCI at real-world hospital settings, CR does not increase short- and long-term mortality, proving to be safe when a staged approach is used.^22^

Although the benefit of a staged strategy following primary PCI has been suggested in several studies including this one, questions remain to be elucidated, such as appropriate timing for staged PCI. In clinical practice, factors such as renal dysfunction, lesion complexity, contrast volume, radiation dose, hemodynamic status, and patient status may influence the decision on optimal timing for revascularization. An electronic survey conducted by the ACC revealed that, although most interventional cardiologists agree to perform CR with a staged approach, their opinions regarding optimal timing for the second PCI vary greatly. Only 22% of respondents performed both first and second interventions at the same hospitalization; most cardiologists recommended waiting at least 15 days for the second procedure.^23^

Despite the evidence and ongoing studies, no study may be able to define a single strategy for patients with STEMI and multivessel CAD. As these patients are heterogeneous, the strategy must be individualized. Undoubtedly, the focus should be on treating the culprit lesion. The decision should ideally be made by a heart team taking anatomical complexity, ventricular function, and patient profile into account in order to reach the best strategy. A complete risk stratification with clinical and angiographic data is crucial for evaluating the patients properly.^24^

The present study has some limitations that should be considered, especially those related to its observational nature. The chance of selection bias cannot be excluded, even though no statistically significant differences were identified regarding the study variables related to the baseline characteristics of patients treated with CR or IR. Also, no change was found in the effect of the strategy on the occurrence of primary outcome due to the factors considered in multivariable analysis, as the intervention strategy was at the operator’s discretion. Moreover, this study included a small number of patients from two centers in southern Brazil and may not be representative of settings in other regions and non-public services.

## Conclusions

In the present study, we used real-world data from clinical practice at two centers in southern Brazil and found that, in patients with multivessel CAD within the context of STEMI undergoing primary PCI, the CR strategy is associated with a significant reduction in primary and secondary outcomes in a 1-year follow-up when compared to the IR strategy. These data should prompt discussion about current clinical and institutional protocols.
